# Inhibiting miR-195-5p Induces Proliferation of Human Corneal Endothelial Cells

**DOI:** 10.3390/ijms241411490

**Published:** 2023-07-15

**Authors:** Mohit Parekh, Tiago Ramos, Stefano Ferrari, Sajjad Ahmad

**Affiliations:** 1 Institute of Ophthalmology, University College London, 11-43 Bath Street, London EC1V 9EL, UK; 2 Fondazione Banca degli Occhi del Veneto Onlus, Via Paccagnella, 11, 30174 Venice, Italy; 3 Moorfields Eye Hospital NHS Foundation Trust, 162 City Rd, London EC1V 2PD, UK; 4 National Institute for Health and Care Research (NIHR) Biomedical Research Centre, Moorfields Eye Hospital NHS Foundation Trust and UCL Institute of Ophthalmology, 162 City Rd, London EC1V 2PD, UK

**Keywords:** cornea, endothelium, microRNA, FECD, extracellular vesicles

## Abstract

Transparency of the human cornea is responsible for clear vision, which is maintained by a monolayer of non-proliferative human corneal endothelial cells (HCEnCs). Dysfunction of these cells can result in irreversible corneal blindness. It is important to identify key factors that limit the proliferation of HCEnCs and thus attempt to reverse them. Extracellular vesicles contain cargo which includes microRNAs (miRNAs) that can modulate a cellular function. In non small cell lung cancer, expression of miR-195-5p has been shown to inhibit proliferation; therefore, we aimed to investigate the inhibitory effect of miR-195-5p in inducing the proliferation of HCEnCs. Human corneal endothelial cell line (HCEC-12) and primary HCEnCs were cultured with miR-195-5p scramble, mimic or inhibitor. Corneal tissues from human cadaveric and FECD donors, and from pigs, mice and rabbits, were used for RT-PCR. miR-195-5p showed an abundance value of 11,363.31 a.u. When normalized against HCEnCs from cadaveric donors, FECD tissues showed a significant upregulation of miR-195-5p (*p* < 0.05) but was significantly downregulated in pig (*p* < 0.001), mouse (*p* < 0.01) and rabbit (*p* < 0.001) CEnCs, which have known proliferative capacity. Proliferation, cell doubling, and wound healing rates were significantly higher when miR-195-5p was inhibited. Inhibiting miR-195-5p showed a significant improvement in viability (HEC staining), decreased cell apoptosis (TdT-dNTP staining) and expression of ZO-1, NA^+^/K^+^-ATPase and Ki-67 markers. Expression of miR-195-5p is found in HCEnCs and FECD cells, which restricts the proliferation of these cells. However, inhibiting miR-195-5p can induce the proliferation of HCEnCs, which opens exciting directions for future research in prolonging FECD pathogenesis by increasing the proliferative capacity of HCEnCs using anti-miR therapy in vivo.

## 1. Introduction

The human cornea is responsible for refracting the incident light and converging it to the retina via a crystalline lens, which is passed on to the optic nerve for clear vision [1]. Human corneal endothelial cells’ (HCEnCs) health is essential to maintain the transparency of the cornea as these cells use pump functions to maintain the balance of fluid between the cornea and the aqueous humor [2,3]. Damage or dysfunction of HCEnCs can lead to fluid accumulation in the cornea leading to edema and visual impairment. It is known that unlike other species, HCEnCs have no mitotic activity in vivo [4,5]. However, they have been recently induced to divide in vitro [6]. As the cell numbers in vivo decrease from as high as 6000 cells/mm^2^ at birth to <2000 cells/mm^2^ during adulthood, the maintenance of these cells is imperative [7,8]. Several pathologies can result in accelerated loss of HCEnCs including viral infections, inflammation, surgical procedures in addition to corneal endothelial dystrophies [9,10,11]. Fuchs’ endothelial corneal dystrophy (FECD) remains one of the leading causes of corneal blindness. FECD is mainly characterized by the loss of cells that leads to chronic and irreversible corneal oedema. Although corneal transplantation is the most common choice of treatment amongst surgeons for the treatment of patients with endothelial dysfunction, the limited availability of donor tissues is not sufficient for the global demand [12]. Alternatives like endothelial cell culture are continuously studied [13]; however, the reliance on donor tissue has not yet dropped significantly. Therefore, options that would reduce the demand of donor corneas need to be identified. Interestingly, unlike humans, primates and feline species, rabbits and pigs have shown proliferation of corneal endothelial cells (CEnCs) in vivo [14,15,16,17]. Pig CEnCs have a limited proliferative capacity compared to rabbits; however,, their proliferative potential remains better than HCEnCs [18]. It would therefore be important to investigate the factors responsible for the proliferation of pig or rabbit CEnCs and compare them with the species that lack proliferation as observed in HCEnCs to open new therapeutic avenues.

In our previous study, we showed that HCEnCs lacked proliferation and migration capacity when the extracellular vesicles (EVs), derived from immortalized human corneal endothelial cells (HCEC-12), were added to the HCEnCs in culture. Several microRNA (miRNAs) were packed in this EV cargo that had different gene expression potential. miRNAs are short, single-stranded, non-coding RNA molecules that are involved in gene expression [19]. The miRNAs degrade or inhibit messenger RNA (mRNA) translation through binding to the complementary RNAs. EVs transfer the cargo from the originating cell to the receiving cell and influence various biological processes such as differentiation, migration, proliferation, etc. [20].

It has been demonstrated that miRNAs act toward biological characteristics including proliferation, cellular apoptosis, migration, and tumorigenesis. In our study, we reported 13 miRNAs with their abundance values. Some of which were found to have key roles in cell cycle (hsa-miR-205-5p; hsa-miR-196b-5p; hsa-miR-122-5p), adherence junction (hsa-miR-196b-5p; hsa-miR-497-5p; hsa-miR-3065-3p; hsa-miR-148b-5p) and p53 signaling pathway (hsa-miR-301a-3p; hsa-miR-196b-5p; hsa-miR-497-5p). hsa-miR-196b-5p was found in all three biological functions, however, its role is mainly known in the progression of cancer cells. It has not been studied in ocular research, thus, needing further investigation [21]. Interestingly, several reports have observed the reduction of miR-195-5p expression in tumors. Jia et al. reported that the overexpression of miR-195-5p could have an application in the inhibition of the development and progression of tongue squamous cell carcinoma [22]. However, the mechanism and target gene of miR-195-5p remains unknown. miR-195-5p has also been shown to inhibit proliferation and induce apoptosis of non-small cell lung cancer cells [22]. As miR-195-5p inhibits the proliferation of cells, we set out to investigate the presence of miR-195-5p in HCEnCs and compare it with rabbit and pig species that have shown proliferative CEnCs in vivo. We further studied the inhibitory effect of miR-195-5p on inducing the proliferation of HCEnCs in vitro which could have a therapeutic potential.

## 2. Results

### 2.1. Cargo Characterization of EVs Showed High Abundance of miR-195-5p

hsa-miR-155 showed the highest abundance value of 99,283.21 a.u., whereas hsa-miR-205 and hsa-miR-205-5p showed the lowest abundance of 21.27 a.u. Relatively, hsa-miR-195-5p was not highly abundant, with a value of 11,363.31 a.u. [21]. As miR-195-5p has been shown to inhibit proliferation in non-small cell lung cancer cells [22], we selected miR-195-5p to further investigate if it was expressed in HCEnCs, which are known to have a non-proliferative characteristic.

### 2.2. miR-195-5p Is Expressed in HCEnCs and HCEC-12 Cell-Derived EVs but Not Found in Pig, Mouse and Rabbit CEnCs

**Figure 1:**
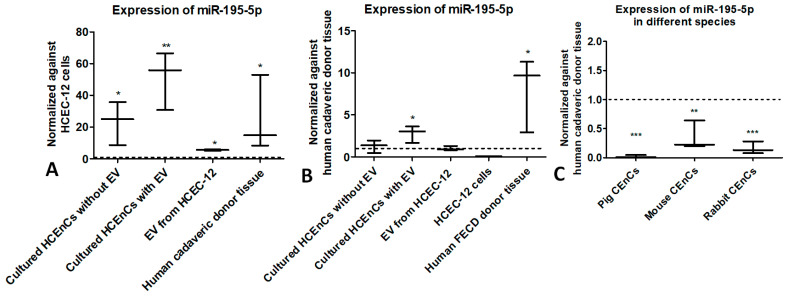
miR-195-5p is expressed in human corneal endothelial cells and cell-derived EVs. (**A**) miR-195-5p was significantly upregulated in cultured HCEnCs with and without EVs, EVs derived from HCEC-12 cells and human cadaveric donor tissue when normalized against HCEC-12 cell lines. (**B**) miR-195-5p showed significant upregulation when HCEnCs were cultured with HCEC-12-derived EVs and human FECD donor tissues when normalized against human cadaveric donor tissues. (**C**) miR-195-5p was significantly downregulated in pig, mouse and rabbit corneal endothelial cells. * *p* < 0.05, ** *p* < 0.01, *** *p* < 0.001.

miR-195-5p was significantly upregulated in cultured HCEnCs without EVs (*p* < 0.05). A further increase in upregulation of miR-195-5p was observed when HCEnCs were cultured with EVs (*p* < 0.01). EVs collected from HCEC-12 cells showed upregulation of miR-195-5p (*p* < 0.05). This suggests that EVs carry a cargo containing miR-195-5p and delivers it to the HCEnCs. Interestingly, we found that miR-195-5p was significantly upregulated in human cadaveric donor CEnCs (*p* < 0.05) when normalized against HCEC-12 cells (Figure 1A). However, when normalized against human cadaveric donor CEnCs, miR-195-5p showed significant upregulation when HCEnCs were cultured with EVs (*p* < 0.05) and human FECD donor tissue (*p* < 0.05) (Figure 1B). Interestingly, miR-195-5p was significantly downregulated in pig (*p* < 0.001), mouse (*p* < 0.01) and rabbit (*p* < 0.001) CEnCs, which are known to have proliferative capacity, to an extent, in vivo (Figure 1C). This suggests that miR-195-5p is readily available in HCEnCs and plays an important role in restricting HCEnC proliferation.

### 2.3. Inhibition of miR-195-5p Increases the Proliferation and Cell Doubling and Reduces the Wound Healing Rate In Vitro

**Figure 2:**
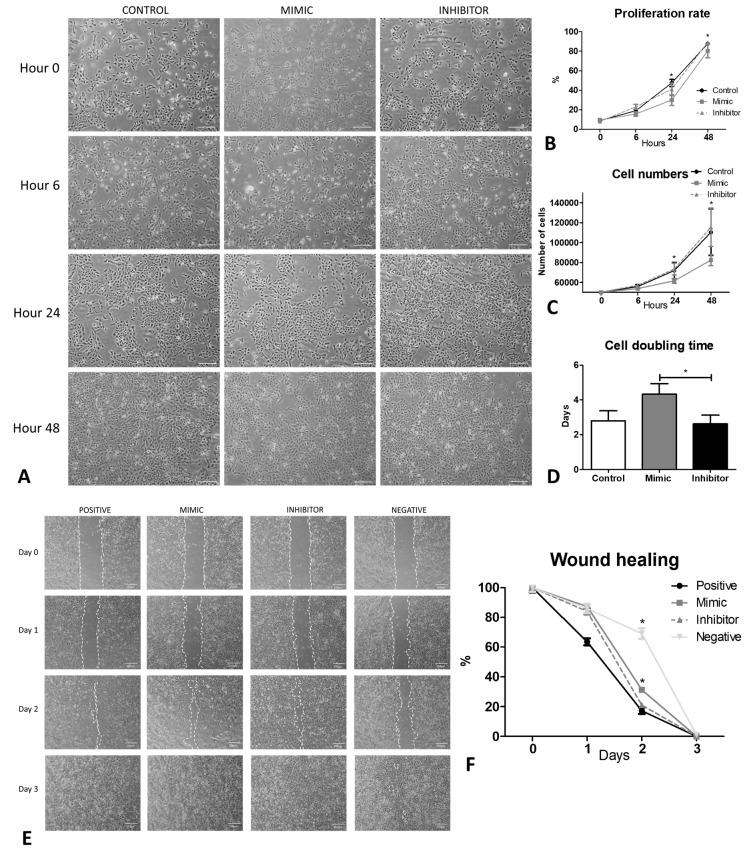
Inhibition of miR-195-5p increases the proliferation rate and decreases the wound healing rate of HCEC-12 cells in vitro. (**A**) cell morphology and growth rate pattern showing increased proliferation rate and cell numbers after treating the HCEC-12 cells with miR-195-5p inhibitor. (**B**) a significantly high proliferation rate was observed following the treatment of cells with miR-195-5p inhibitor with (**C**) increased cell numbers in 24 h compared to the mimic. (**D**) cell doubling time of the cells treated with the inhibitor was significantly less compared with the mimic. (**E**) wound healing response after inhibiting the cells with miR-195-5p showed (**F**) a significant healing response on day 2. * *p* < 0.05. Scale bar: 250 µm.

Inhibition of miR-195-5p did not show abnormal morphology (Figure 2A) with an increased proliferative capacity (Figure 2B) and cell numbers (Figure 2C) compared with mimic within 24 h (*p* < 0.05). The cell doubling time was significantly reduced from 4.3 ± 0.6 days as observed from cells treated with mimic to 2.6 ± 0.5 days from miRNA inhibition group (*p* < 0.05) (Figure 2D). A significantly early wound healing was observed by day 2 (*p* < 0.05) when the cells were inhibited with miRNA inhibitor (Figure 2E,F).

### 2.4. Inhibiting miR-195-5p Increases the Cell Viability and Reduces Apoptosis and Cell Area

**Figure 3:**
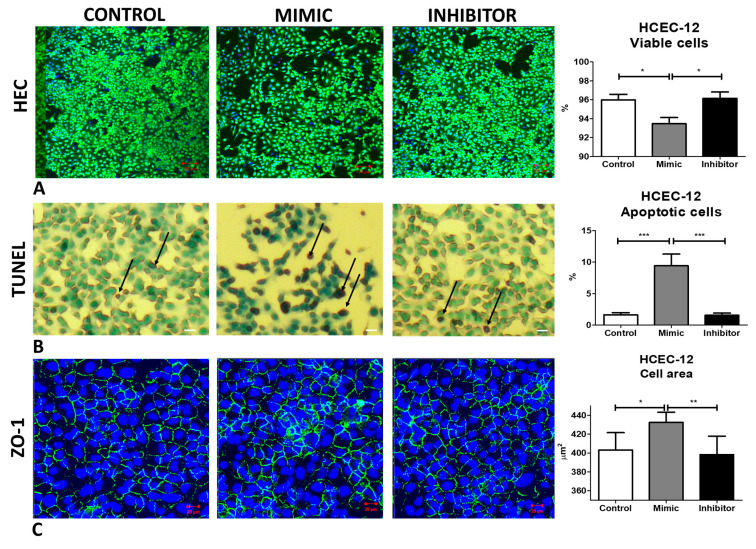
Inhibiting miR-195-5p increases the cell viability and reduces apoptosis and cell area. (**A**) HEC staining showed a significantly higher viability (Calcein-AM positivity) of HCEC-12 cells. (**B**) Apoptosis was rescued when HCEC-12 cells were treated with miR-195-5p inhibitor as shown by TUNEL assay (marked with black arrow with methyl green as counter stain). (**C**) increased cell area was observed from the cells treated with miR-195-5p mimic but not with miR-195-5p inhibitor as observed by ZO-1 (Zonula occludens 1) staining. Triple labelling showed the presence of Ethidium Homodimer stained in red representing dead cells, Hoechst in blue representing nuclei and Calcein AM in green marking viable cells. * *p* < 0.05, ** *p* < 0.01, *** *p* < 0.001. Scale bar: (**A**) 100 µm, (**B**,**C**) 20 µm.

A significant drop in the viability of cells (Calcein AM positive) was observed when the cells were treated with mimic (93.4 ± 0.6%) compared to inhibitor treated cells (96.2 ± 0.7%) (*p* < 0.05) (Figure 3A). miR-195-5p mimic treated cells also showed a significantly higher percentage of apoptotic cells (marked in black arrow) (9.4 ± 1.9%) compared to inhibitor treated cells (1.6 ± 0.4%) (0 < 0.001) (Figure 3B), as observed using TdT dNTP assay. ZO-1 was expressed, and the staining showed a significantly higher cell area indicating polymegathism when the cells were treated with mimic (432.5 ± 10.2 um^2^) compared to the inhibitor treated cells (398.3 ± 21.7%) (*p* < 0.01) (Figure 3C). No difference was observed in hexagonality or polymorphism from either group (Supplementary Figure S1A,B).

### 2.5. Inhibition of miR-195-5p Increases the Proliferation Rate and Wound Closure of HCEnCs

**Figure 4:**
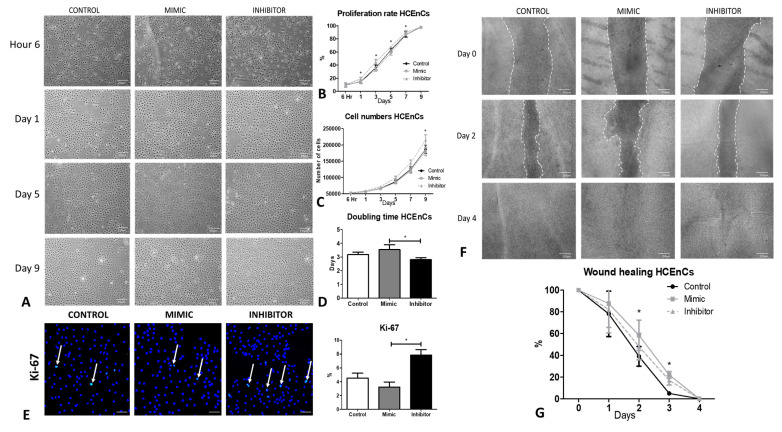
Inhibition of miR-195-5p increases the proliferation rate and Ki-67 positivity and decreases the wound healing rate of HCEnCs. (**A**) Cell morphology and growth rate showing improved proliferation rate after treating HCEnCs with miR-195-5p inhibitor at different time points. (**B**) A significantly high proliferation rate was observed following the treatment of cells with miR-195-5p inhibitor with (**C**) increased cell numbers in 9 days compared to the mimic. (**D**) Cell doubling time of the cells treated with the inhibitor was significantly less compared with the mimic. (**E**) Ki-67 positive cells (white arrows) were significantly higher in the HCEnCs treated with miR-195-5p inhibitor. (**F**) Wound healing response after inhibiting the cells on human cadaveric donor tissues with miR-195-5p showed (**G**) a significantly low healing response at day 2 and 3 after treating the cells with miR-195-5p mimic compared to the inhibitor. * *p* < 0.05. Scale bar: (**A**,**F**) 250 µm, (**E**) 50 µm.

Treatment of HCEnCs with miR-195-5p mimic showed enlarged cell morphology by day 5 (Figure 4A). A significantly high proliferative capacity (*p* < 0.05) (Figure 4B) was observed from day 1 when HCEnCs were treated with miR-195-5p inhibitor. Although the cell numbers gradually increased, a significant difference was only observed on day 9 when the cells were treated with the inhibitor (Figure 4C) compared with the mimic (*p* < 0.05). The cell doubling time was significantly reduced from 3.5 ± 0.3 days, as observed from cells treated with mimic, to 2.8 ± 0.1 days from miR inhibition (*p* < 0.05) (Figure 4D). To support the proliferation data, the cells were stained with Ki-67, and a significantly high expression of Ki-67 was observed following the treatment with miR inhibitor (7.9 ± 1.3%) compared with the mimic (3.2 ± 1.3%) (*p* < 0.05) on day 9 (Figure 4E). Scratch assay on human donor tissues (Figure 4F) showed a significant wound healing response on day 2 and 3 (*p* < 0.05) when the tissues were inhibited with miRNA inhibitor (Figure 4G) compared to the mimic. Note: The culture medium contains growth factors for cell culture required to maintain these tissues; hence, the tissues treated with mimic also showed complete wound healing by day 4. However, the wound healing was significantly delayed when the cells were treated with the mimic compared with the inhibitor.

### 2.6. Inhibiting miR-195-5p Increases the Cell Viability and Reduces Apoptosis and Cell Area of HCEnCs

**Figure 5:**
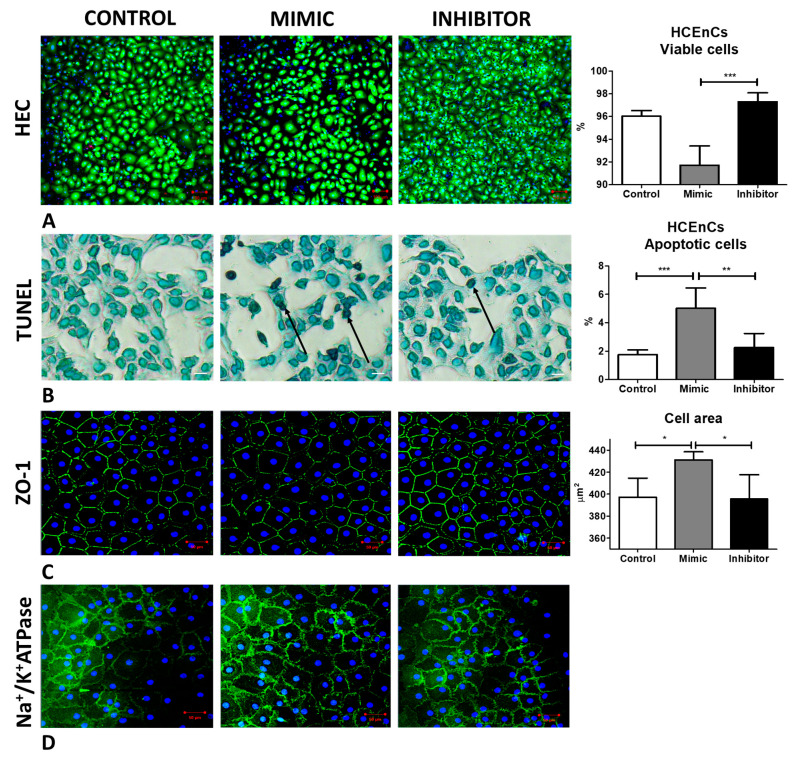
Inhibiting miR-195-5p increases the cell viability and reduces apoptosis and cell area; however, it does not change the pump functions. (**A**) HEC staining showed a significantly higher viability (Calcein-AM positivity) of HCEnCs when treated with miR-195-5p. (**B**) Apoptosis was rescued when HCEnCs were treated with miR-195-5p inhibitor as shown via TUNEL assay (marked with black arrow with methyl green as counter stain). (**C**) Increased cell area was observed on the cells treated with miR-195-5p mimic but not with miR-195-5p inhibitor as observed by ZO-1 (Zonula occludens 1) staining. (**D**) Na^+^/K^+^-ATPase pump function was not affected by miR-195-5p mimic. * *p* < 0.05, ** *p* < 0.01, *** *p* < 0.001. Scale bar: (**A**) 100 µm, (**B**–**D**) 50 µm.

A significant drop in the viability of cells (Calcein Am positive) was observed when the cells were treated with the mimic (91.7 ± 1.7%) compared to inhibitor-treated cells (97.3 ± 0.8%) (*p* < 0.001) (Figure 5A). miR-195-5p treated cells also showed a significantly higher percentage of apoptotic cells (marked in black arrow) (5.1 ± 1.4%) compared to the inhibitor-treated cells (2.3 ± 0.9%) (*p* < 0.01) (Figure 5B) as observed using TdT dNTP assay. ZO-1 staining showed a significantly higher cell area, indicating polymegathism, when the cells were treated with the mimic (359.9 ± 174.4 um^2^) compared to the inhibitor-treated cells (330.1 ± 161.5%) (*p* < 0.05) (Figure 5C). No difference was observed in hexagonality or polymorphism from either group (Supplementary Figure S1C,D). There was no difference observed between the cells treated with the mimic or inhibitor in terms of pump functions as observed by Na^+^/K^+^ ATPase staining (Figure 5D).

## 3. Discussion

The corneal endothelium is a monolayer of neural crest–derived cells with limited regenerative potential. However, it is still not clear why these cells do not possess a regenerative capacity. Endothelial cells are continuously lost during the aging process and an accelerated loss is observed even after uncomplicated surgeries. Identifying the factors that could expedite controlled proliferation of HCEnCs upon severe cell loss would be ideal to avoid surgical intervention. In our previous study, we showed HCEnCs lacked proliferation and migration capacity when the extracellular vesicles (EVs) derived from HCEC-12 cells containing several miRNAs were cultured with HCEnCs in vitro [21]. miRNAs play an important role in general growth and development, or disease progression [23].

In our study [21], hsa-miR-155, a master regulator of inflammation, showed the highest abundance value. Although not found in extreme abundance, hsa-miR-195-5p was relatively high amongst those expressed in our EV cargo. miR-195-5p is known to inhibit proliferation in non small cell lung cancer cells [22]; therefore, we set our investigation towards increasing the proliferation rate of HCEnCs by inhibiting this miRNA without losing the cellular function. As the HCEC-12 cells are immortalized, it is likely that miR-195-5p expression in these cells get modulated as these cells are proliferative. miR-195-5p expression was increased when the cells were cultured with EVs. EVs are released by the cells; therefore, if the miRNA is present in the cell, it would be encapsulated within the EVs. These EVs then travel to the neighboring cells and deliver the cargo and play the role of messengers. Human cadaveric donor cells showed increased expression of miR-195-5p, indicating the need of investigation of this miRNA towards the non-proliferate nature of HCEnCs. In addition, FECD cells derived from the donor tissue showed a significant upregulation of miR-195-5p, indicating why the diseased cells have an extremely low proliferative capacity. However, the mechanism of miR-195-5p upregulation in FECD is still unknown. FECD cells are diseased and do not proliferate; the upregulation of miR-195-5p could be one of the potential factors that may lead towards the treatment of FECD. Interestingly, miR-195-5p was significantly downregulated in pig, mouse and rabbit CEnCs, which have a proliferative capacity, further indicating the important role of miR-195-5p in proliferation. In addition, we observed that by inhibiting miR-195-5p, HCEC-12 and HCEnCs showed increased proliferative capacity and cell numbers with a significantly early wound-healing response. This observation suggests that miR-195-5p may play an important role in therapeutic intervention towards the proliferation of HCEnCs. The cell area decreased due to proliferation after inhibiting miR-195-5p as the cells became compact, and the cells expressed the hallmark biomarkers of HCEnCs, such as ZO-1 and Na^+^/K^+^-ATPase. This means that HCEnCs can be induced with proliferative capacity without hampering its functional activity by inhibiting miR-195-5p.

miRNA has a key role in multiple biological metabolic processes. In fact, it has been observed that a low expression of miR-195-5p can lead to tumors and may be associated with cancer development [24,25]. A study by Shao et al. reported that miR-195-5p is downregulated in cervical cancer serum and tissue samples. This could increase cell invasiveness by regulating the target gene cyclin D1, leading to a potential therapeutic target [26]. In our study, we observed a significantly higher expression of proliferating cell cycle-associated nuclear antigen (Ki67), which is a protein antigen related to cell division and proliferation. The expression of Ki-67 is closely related to cell proliferation, and important for the regulation of the cell cycle [27]. In our study, we found that inhibiting miR-195-5p increases the expression of Ki67, which was also shown in a study by Liu et al. [28]. We further observed that the viability of cells following the treatment with the miR-195-5p mimic was reduced. This phenomenon could also be a result of increased apoptosis as observed from the TdT-dNTP assay. Inhibiting miR-195-5p significantly reduced the apoptotic cells thus, opening new pathways for research to increase the health of CEnCs by inhibiting miR-195-5p. miR-195-5p can have multiple pathways and different roles in different cell types. Hence, it must be carefully validated before being used as a potential therapy. In cancer cells, promoting apoptosis would lead to the inhibition of cancer cells’ invasive ability. However, it would work otherwise in HCEnCs, where the cells must be induced with proliferative capacity; hence, inhibiting miR-195-5p would become a sensible therapeutic approach.

It is also true that the activity of miR-195-5p alone could have a specific effect, but when in combination with other miRNAs, it may have a deleterious or compensatory effect. Multiple miRNAs, such as miR-34a and miR-378, have shown to be associated with phenotypic profiling of cultured HCEnCs [29], whereas miR-34-a-5p, miR-24-3p, miR-34c-5p, miR-34a and miR-184 have been observed in aqueous humor metabolites suggesting their ability to maintain tissue hydration [30]. Critical miRNAs found for the regulation of the aging of corneal endothelial cells in mice include miR-29c, miR-34c, miR-124, miR-695 and miR-32 [31]. MiR-695, miR-31, miR-190, miR-183, miR-182 and miR-194 are the most significantly downregulated, whereas miRNAs, and miR-34c and miR-124 have shown to be significantly upregulated. In the HCEC cell lines, the effect of miR-30c-1 in cell propagation has been shown. It was found that cell-aging effects of TGF- β1 were converted by miR-30c-1, making it a potentially feasible treatment molecule for the regeneration of HCEnCs [32]. Three miRNAs from the miR-29 family (miR-29a-3p, miR-29b-2-5p and miR-29c-5p) were significantly downregulated. When FECD samples were compared to normal samples, miR-29a was found to be the most deregulated miRNA [33]. miRNAs found causing the regulation of the aging of mice CEnCs include miR-29c, miR-34c, miR-124, miR-695 and miR-32. Another study showed miR-302a’s capacity for eliminating IFN-γ-induced senescence and cellular damage through oxidative and ER stress regulation and also promoted the proliferation of HCEnCs [34]. To our surprise, miR-195-5p, which is relatively abundant in the HCEnCs and FECD cells, has never been described before. Although this miRNA is not found in extreme abundance in HCEnCs, its role in cell proliferation seems clear. Hence, we also recommend screening those miRNAs that may not be in abundance but could have a significant impact on the cell processes.

## 4. Materials and Methods

### 4.1. Ethical Statement

Human donor corneas unsuitable for transplantation were obtained from Fondazione Banca degli Occhi del Veneto (FBOV, Venice, Italy). The tissues were utilized and discarded as per the Human Tissue Authority (HTA, UK) guidelines. All the experiments were approved by the UCL ethics committee (10/H0106/57-2011ETR10) and were performed in accordance with the Declaration of Helsinki. The corneas from pigs and rabbits were obtained from whole eyes shipped by a local abattoir and the mice corneas were obtained from the UCL animal facility when the mice were used for other research and the cornea was not required, thus not qualifying for any special animal handling approval/ARVO guidelines for animal handling for this research.

### 4.2. Extraction of Extracellular Vesicles and Cargo Characterization by Next Generation Sequencing

The protocol for EV extraction was described earlier by Parekh et al. [21] and Ramos et al. [35]. Briefly, upon confluence, HCEC-12 cells were serum starved for 72 h. The medium was centrifuged at 500 rpm for 5 min at 4 °C (centrifuge 5417, Eppendorf, Hamburg, Germany) followed by 2000 rpm for 10 min at 4 °C, sequentially. The resulting supernatant was filtered through a 0.22 micrometer filter (Merck Millipore, Burlington, MA, USA) and transferred to OptiSeal tube (Beckman Coulter, Brea, CA, USA). The supernatant was ultracentrifuged at 49,000 rpm (100,000× *g*) using a 90Ti fixed angle rotor (Beckman Coulter, Brea, CA, USA) for 2 h at 4 °C. The pellet was re-suspended in PBS, followed by a second ultracentrifugation. The final pellet was re-suspended in the media, PBS or Trizol at −80 °C for further experiments. For the measurement of small RNA concentration (Agilent Bioanalyzer Small Assay using Bioanalyzer 2100 Expert instrument—Agilent Technologies, Santa Clara, CA, USA), 1 μL of total EV-RNA was utilized. NGS libraries were generated as described earlier [21,35], and the sequencing was performed on Illumina NextSeq 500 platform. DEseq was used for abundance determination and differential expression. TarBase v7.0 of KEGG (mirPath v.2, Diana tools) was used for pathway analysis.

### 4.3. Primary Cell Culture

The endothelial layer of corneal tissue from human cadaveric donors was peeled and digested in 2 mg/mL collagenase Type 1 (Thermo Fisher Scientific, Rochester, NY, USA) followed by centrifugation for 5 minutes at 1000 rpm. The pellet was re-suspended in TrypLE Express (1×) (Life Technologies, Monza, Italy) to dissociate single cells and centrifuged. The cells were re-suspended in 1 mL of the cell culture medium (1:1 Ham’s F12:M199 (Sigma-Aldrich, Gillingham, Dorset, UK)), 5% FBS, 20 µg/mL ascorbic acid (Sigma-Aldrich), 1% Insulin Transferrin Selenium (Gibco, Rochester, NY, USA), 10 ng/mL recombinant human FGF basic (Gibco), 10 µM ROCK inhibitor (Y-27632) (Miltenyi Biotech, Charlestown, MA, USA) and 1% PenStrep (Sigma-Aldrich) [36]. The HCEnCs were counted using a haemocytometer, and the number of plated cells was recorded. Lab-Tek II chamber slides were coated with 50 µL Fibronectin Collagen (FNC) coating mix (US Biological Life Sciences, Salem, MA, USA) for at least 15 minutes at 37 °C and 5% CO_2_. The cells were plated, and the medium was replaced every alternate day until confluence.

### 4.4. Cell Culture with EVs or Treated with miR-195-5p Scramble, Mimic or Inhibitor

The cells (HCEC-12 and HCEnCs) were treated uniformly. For the EV study, approximately 10 × 10^7^ particles/mL (obtained from NanoSight) EVs were added on roughly 50,000 cells (1:100 cell/EV ratio) and followed by the collection of the cells in Trizol for RT-PCR. To determine the effect of miR-195-5p, the cells were transfected using the manufacturer’s protocol with miR-195-5p scramble (control), mimic or inhibitor (Applied Biological Materials Inc., Richmond, BC, Canada).

### 4.5. Hoechst, Ethidium Homodimer and Calcein AM (HEC) Staining to Determine Live/Dead Cells

Following 80% confluency after the treatment of cells with control, mimic or inhibitor, the cells were washed with PBS and treated with 5 µL of Hoechst 33342 (H) (Thermo Fisher Scientific), 4 µL of Ethidium Homodimer EthD-1 (E) and 2 µL Calcein AM (C) (Live/Dead viability/cytotoxicity kit, Thermo Fisher Scientific) mixed in 1 mL of PBS. The cells (HCEC-12 and HCEnCs) were incubated with 100 µL of the final solution at RT in dark for 30 min, followed by a single washing step. The cells were mounted and analyzed using Zeiss LSM 700 confocal microscope (Carl Zeiss, Cambridge, UK) [37].

### 4.6. Cell Apoptosis Using Terminal Deoxynucleotidyl Transferase Deoxyuridine Triphosphate Nick-End Labelling Assay

Cell apoptosis assay was performed according to the manufacturer’s instructions for TACS2 terminal deoxynucleotidyl transferase (TD) diaminobenzidine (DAB) in situ apoptosis detection kit (Trevigen, Gaithersburg, MD, USA) as described earlier [38]. A separate set of samples were induced for apoptosis using TACS nuclease to validate the protocol. The samples were viewed using a standard inverted light microscope (Nikon Eclipse, TS100, Nikon, Surrey, UK). The apoptotic cells were manually counted with an average of 5 readings per sample for statistical analysis.

### 4.7. Immunostaining of Proliferation Marker (Ki-67), Tight Junctions (Zonula Occludens-1; ZO-1) and Pump Functions (Na^+^/K^+^ ATPase)

Upon confluence, the cells were washed with PBS and fixed in 4% paraformaldehyde (PFA) at RT for 20 min. The cells were permeabilized with 0.25% Triton X-100 for 15 min and blocked with 10% goat serum for 1 h at RT. The samples were incubated overnight at 4 °C with primary antibodies anti-Ki67 (1:200), anti-ZO1 (1:200) and anti-Na^+^/K^+^ ATPase (1:50). The cells were incubated with goat anti-mouse/anti-rabbit conjugated with Alexa Flour 488 secondary antibody for 1 h at RT. After each step, the cells were washed thrice. The cells were mounted with Vectashield containing DAPI. Expression of the markers were examined using LSM 700 confocal microscope (Carl Zeiss).

### 4.8. RNA Isolation and Quantitative Real-Time PCR for miR-195-5p

The cells and EV pellet were lysed in 450 µL of Trizol and the small RNA fractions were extracted using mirVana™ miRNA Isolation Kit (ThermoFisher, Waltham, MA, USA) according to the manufacturer’s instructions. The RNA purity and quantity was assessed using NanoDrop (ND1000 Nanodrop Technologies, Wilmington, DE, USA). TaqMAn™ MicroRNA Reverse Transcription kit was used as per the recommended procedure (ThermoFisher). Reverse transcription reactions were performed on a MasterCycler Gradient 5331 (Eppendorf, Hamburg, Germany) with the following conditions: 16 °C for 30 min, 42 °C for 30 min, 85 °C for 5 min and 4 °C on hold. TaqMan^®^ Fast Universal PCR Master Mix (2×) no Amperase^®^ UNG was used for RT-PCR according to the manufacturer’s instructions. RT-PCR was performed in a QuantStudio 6 Flex Real-Time PCR System (ThermoFisher) at of 95 °C for 10 min, followed by 40 cycles of 95 °C for 10 s and 60 °C for 1 min. hsa-miR-195-5p expression (ThermoFisher) was analyzed against the expression of the small-nucleolar RNA RNU44 (ThermoFisher). The fold increase was calculated using ΔΔCt method [39].

### 4.9. Analysis

#### 4.9.1. Proliferation Rate, Cell Doubling and Time

The proliferation rate was measured every alternate day using an in-built reticule (10 × 10) attached to an inverted microscope (Nikon Eclipse TS100; Nikon). The number of endothelial cells/mm^2^ were counted using the same reticule determined by counting the number of blocks filled by the cells every alternate day represented as percentage of proliferation in the given area. Cell doubling numbers and time were also calculated similarly.

#### 4.9.2. Wound Healing Rate

Upon confluence on a 12-well culture plate, HCEC-12 cells were scratched centrally using a 10 μL pipette tip. Central straight scratch was created using a 1 mL pipette tip on human corneal donor tissue to determine the wound healing rate ex vivo. The wound closure was monitored every 24 h following the transfection of cells with the scramble, mimic or inhibitor and the rate of closure was determined by ImageJ.

#### 4.9.3. Viability and Ki-67 Positivity

All the measurements and data analysis were performed using ImageJ (FIJI) bundled with 64-bit Java 1.8.0 112. The viability of cells was measured as the number of Calcein AM positive cells compared with the number of Hoechst positivity. The green and blue channels were split and converted to binary image. Following the thresholding to avoid false positive values, the image was subjected to particle analysis which provided total number of blue (nucleus) and green (Calcein AM) cells. Similarly, for Ki-67 analysis, the image was split into blue and green channels, and the analyzed values were obtained to convert them into percentage for statistical analysis.

#### 4.9.4. Hexagonality and Cell Area

The ZO1 positive images were converted to overlay masks using pre-determined macroinstructions to define the parameters of both hexagonality and polymorphism as described earlier [40]. Once the macros provided the binary image with precise borders, the cells were counted manually depending on the cell structure that comprised 6 borders for hexagonal cell or <4 borders for severe polymorphism. Cell area (µm^2^) was measured by marking the borders of the cells using a free-hand tool followed by an area measurement in ImageJ. 

### 4.10. Statistical Analysis

A one-way ANOVA test and post hoc Bonferroni’s test with a significance level of α = 0.05 (95% confidence intervals) were used to compare the data between the control, mimic and inhibitor using GraphPad Prism 8.0.1 software. A probability value of <0.05 was deemed statistically significantly different. All the experiments were performed at least in triplicate.

## 5. Conclusions

In conclusion, we identified a unique miRNA that is expressed in the corneal endothelium and may facilitate functional changes towards increasing the proliferative potential of HCEnCs when inhibited. However, we have not checked the off-target effects of this miRNA in this pilot study. Fluctuations in the expression of this specific miRNA or in combination with other miRNAs may result in different mechanisms leading to functional modulations. Therefore, our results identify exciting directions for future research, especially as this miRNA is also found in FECD samples. This could potentially open a new avenue for research in the field of FECD pathogenesis or treatment. Further studies are needed to investigate the regulatory mechanisms of miR-195-5p. Understanding miRNA expression and interactions in a tissue such as the cornea may help in understanding the basic and pathophysiological processes of disease progression. Anti-miR-195-5p therapy is not limited to preventing FECD; it could also be used for techniques like Descemet stripping only, where the endothelial wound healing mainly depends on the peripheral endothelial cell migration and proliferation [41] or to treat bullous keratopathy.

## Data Availability

The datasets presented in this article are not readily available because they are in the internal server of UCL Institute of Ophthalmology. Requests to access the datasets should be directed to Sajjad Ahmad.

## Supplementary Materials

The following supporting information can be downloaded at: https://www.mdpi.com/article/10.3390/ijms241411490/s1.
