# Supplementary material for: Inhibiting miR-195-5p Induces Proliferation of Human Corneal Endothelial Cells
Source: Int J Mol Sci. 2023 Jul 15;24(14):11490. doi: 10.3390/ijms241411490 (PMC10380751; doi:10.3390/ijms241411490)
Supplement: Supplementary file 1 [file ijms-24-11490-s001.zip › ijms-2474813-supplementary.pdf]

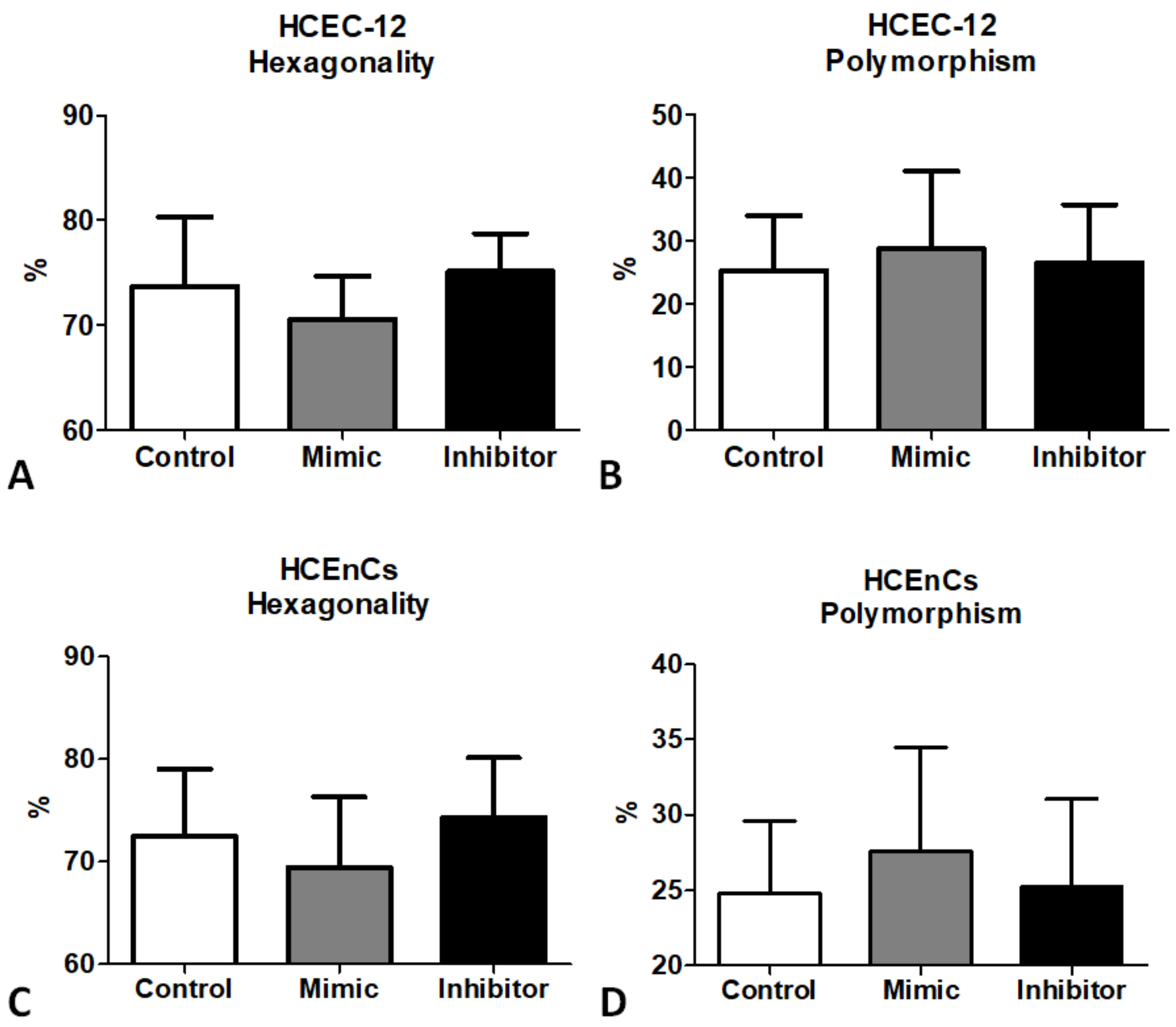

Supp. Figure S1: Hexagonality and polymorphism, **(A,B**) no difference was observed in hexagonality or polymorphism from either group (control, mimic or inhibitor) in HCEC-12 lines or (**C,D**) in HCEnCs.
